# A Comparative Analysis of the Use of Low-Calorie and No-Calorie Sweeteners in Food Products Without and With Added Sugar on the Polish Market

**DOI:** 10.3390/nu17111899

**Published:** 2025-05-31

**Authors:** Aleksandra Kołodziejczyk, Justyna Nowak

**Affiliations:** 1 Department of Food Technology and Quality Assessment, Department of Dietetics, Faculty of Public Health in Bytom, Medical University of Silesia, 41-900 Bytom, Poland; 2 Department of Metabolic Disease Prevention, Faculty of Public Health in Bytom, Medical University of Silesia, 41-902 Bytom, Poland

**Keywords:** Poland, low-calorie sweeteners, no-calorie sweeteners, products without added sugar, added sugars, additives

## Abstract

Background/Objectives: The growing interest of consumers in reducing their sugar consumption has led to an increase in the popularity of food products containing low- and no-calorie sweeteners as an alternative to sugar. The aim of this study was to comparatively analyze the presence of these substances in food products available on the Polish market. Methods: The analysis was performed on the labels of 1278 food products available on the Polish market in the second and third quarters of 2023. The products were divided into two groups: those with added sugar and those without added sugar. The presence of added sugars, intense sweeteners, polyols and steviol glycosides was assessed. Results: This study showed that 33.9% of the products without added sugar and 20% of the products containing sugar contained sweeteners. In sugar-free products, polyols dominated (22.9%), while in products with sugar, intense sweeteners were most often used, mainly sucralose (13.6%). Significant differences (*p* < 0.0001) were observed in the presence of polyols and intensive sweeteners between the analyzed groups. Conclusions: Products without added sugar more often contain low- and no-calorie sweeteners than products with sugar, which suggests their greater use in this category. Repeated use of the same sweeteners, in the absence of labeling, may lead to cumulative consumption, potentially causing acceptable levels to be exceeded. Further research is needed to assess this risk and develop strategies to reduce their excessive consumption.

## 1. Introduction

The increase in the incidence of chronic non-communicable diseases such as type 2 diabetes, cardiovascular diseases, obesity and metabolic syndrome in recent decades has become a serious challenge for public health in developed countries [1,2]. Research evidence indicates that excessive weight gain is a major risk factor for the development of type 2 diabetes and cardiovascular diseases [2,3,4]. Research suggests that reducing sugar intake in the context of weight control is effective when combined with an overall reduction in total calorie intake, which leads to an improvement in energy balance and helps maintain a healthy body weight [5,6,7]. In response to these observations, public health institutions in many countries are taking actions to reduce sugar consumption, especially for products of low nutritional value, which mainly provide energy without supporting consumers’ health [2,8].

An increase in public awareness of the negative impact of sugar on health has contributed to the implementation by public health authorities of a number of policies aiming to reduce its consumption. These actions include, among others, the promotion of informed dietary choices and reformulation of the composition of food products [8,9]. In response to changing consumer expectations and legal regulations, food manufacturers are increasingly eliminating sugar from products or replacing it with low- and no-calorie sweeteners (LNCSs) [10]. These sweeteners, which are among the most frequently studied food additives, provide a sweet taste with a much lower energy value compared to that of sugar, which helps reduce the caloric value of these products and limits their impact on blood glucose levels [5,10,11,12]. Although their use is strictly controlled by European regulations and the most commonly used substances—such as aspartame, sucralose, erythritol and stevia—have been recognized as safe by international regulatory bodies [10,12,13,14], there are still discussions about their long-term health effects [1]. In addition, there are concerns about the increased use of LNCSs and the potential risk of exceeding the acceptable daily intake (ADI) due to a lack of detailed information on their amounts in product labeling [10,15,16].

The growing popularity of low-sugar products and the widespread use of LNCSs in the food industry emphasize the need to monitor their presence on the market and analyze the types of sweeteners used. This information is fundamental for assessing the risk associated with their consumption and developing effective health policies [9]. The aim of this study was to analyze the frequency and types of low- and no-calorie sweeteners in food products available on the Polish market, with particular emphasis on the differences between products containing sugar and those without it.

## 2. Materials and Methods

### 2.1. Sample Selection

This study was based on an analysis of the sweeteners declared on the labels of food products available on the Polish market. A total of 1278 food products were selected for this analysis. The sample was randomly selected from four online stores and three stationary retail outlets, covering both large retail chains and smaller local stores, to obtain a cross-sectional set of products available on the market. The data were collected during the second and third quarters of 2023.

The selection followed a stratified approach aiming to ensure adequate representation of the product categories in which low- and no-calorie sweeteners (LNCSs) are commonly used. The selection criteria were informed by the previous literature, market availability, and label accessibility.

Products were included in this study if they met the following criteria:

(1) The product was available for retail sale in Poland during the study period;
(2) The product label contained clear information about the presence or absence of added sugar and/or sweeteners;
(3) The product belonged to one of the food categories described below.

### 2.2. Product Classification

**Figure 1:**
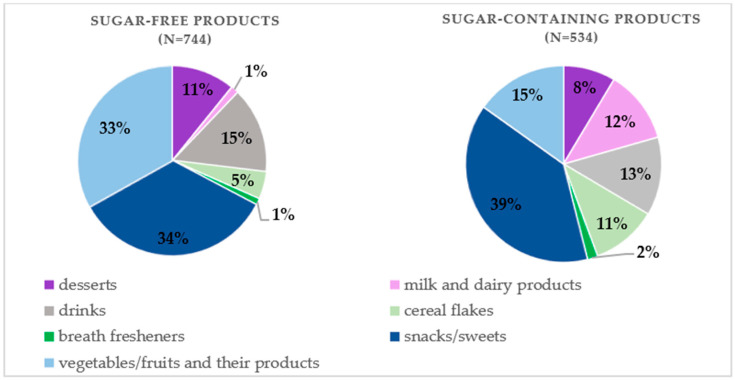
Percentage share of products in each category.

Products were divided into two main groups: products labeled as “without added sugar” (N = 744) and products containing sugar (N = 534). The “no added sugar” category includes products which, in accordance with Regulation (EU) No. 1333/2008 [17], do not contain added mono- or disaccharides but may contain naturally occurring sugars, e.g., from fruit or milk. Product categories were defined based on the practical retail classification and included desserts, milk and dairy products, beverages, cereals, breath-freshening products, snacks/sweets, and vegetables/fruits and dairy products. The proportion of products within each category is presented in Figure 1. To ensure the anonymity of the results, each product was assigned a unique numerical identifier. The data was organized in a database using Microsoft Excel software, which facilitated efficient processing and statistical analysis of the dataset.

This study focused on comparing the formula composition of products labeled as “without added sugar” with that of products containing sugar, with particular emphasis on declared sweeteners. The analyzed substances were divided into four main groups: added sugars (sucrose, monosaccharides, starch hydrolysates, fruit extracts and honey), intensive artificial sweeteners (acesulfame K, aspartame, cyclamic acid and its salts, saccharin and its salts, sucralose, thaumatin, neohesperidin DC, neotame and the salts of aspartame and acesulfame), steviol glycosides and polyols (sorbitol, mannitol, xylitol, erythritol, maltitol, lactitol and isomalt). Each product was assessed based on the presence of these substances, as well as the number of different sweeteners that could be present in one product at the same time. Table 1 presents detailed assessment criteria regarding the number of sweeteners in the individual products.

This study did not require the consent of the Bioethics Committee because it was not a medical experiment and its scope was limited to the analysis of data publicly available on food product labels.

### 2.3. Data Processing

The obtained results were statistically analyzed using STATISTICA 13.3 (StatSoft Polska, Poland). In the case of qualitative data, the occurrence of individual variants was counted and presented in the form of numbers and percentages. The Shapiro–Wilk test was used to assess the normality of the data distribution. Comparisons between groups were performed using tests of the significance of differences. In the case of qualitative data, a test of the significance of differences between two structure indicators was used, which allowed for an assessment of the differences between product categories. Relationships between variables were analyzed using the Chi-2 test. The adopted level of significance α = 0.05 meant that *p* values below 0.05 were considered statistically significant, indicating the occurrence of significant differences between the variables studied.

## 3. Results

Among all products analyzed, low-calorie and no-calorie sweeteners (LNCSs) were more frequently found in products labeled as “no added sugar” than in products containing sugar. Polyols were the most abundant group in the sugar-free products, while artificial sweeteners dominated in products with added sugar. In the group of products containing sugar in addition to sugar, glucose syrup was the most common (17.1%), and sucralose was the most frequently detected intense artificial sweetener in both groups. The most frequently detected polyols in the group of products without added sugar were maltitols (10.1%), while sorbitol dominated in products containing sugar (6%).

Products labeled as “no added sugar” contained artificial sweeteners and polyols significantly more frequently, while the difference in the use of steviol glycosides was not statistically significant. The results are presented in Table 2.

A further analysis by product category revealed statistically significant differences in the distribution of sweeteners. The analysis showed that in each of the analyzed groups of products without added sugar, there was at least one product containing intensive artificial sweeteners. The percentage of sugar-free products containing intensive artificial sweeteners was significantly higher (*p* < 0.05) in categories such as desserts; milk and dairy products (*p* = 0.01); breath-freshening products; and sweets and snacks. In the group of products with added sugar, intensive artificial sweeteners were significantly more common in groups such as beverages (*p* = 0.0007) and vegetables, fruits and their products.

Polyols were particularly prevalent in the sugar-free breath-freshening products (100%), and their use was significantly more frequent in groups of products without added sugar such as desserts; cereals; breath-freshening products; sweets and snacks; and vegetables, fruits and their products. The above results are presented in Table 3.

**Figure 2:**
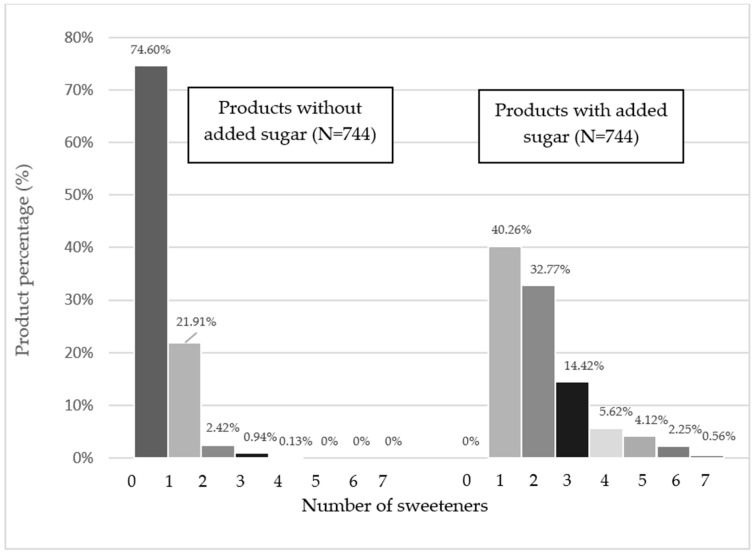
A percentage comparison of the products in terms of the number of sweeteners used in the analyzed products.

An analysis of the number of sweeteners per product revealed that the majority of products labeled “without added sugar” did not contain any sweeteners, whereas products with added sugar most frequently contained a single sweetener. The use of multiple sweeteners was considerably more prevalent among sweetened products, with some items containing up to seven distinct sweeteners. The above results are presented in Figure 2.

## 4. Discussion

The growing interest of consumers in reducing the consumption of added sugars has contributed to the growing popularity of food products containing low- and no-calorie sweeteners (LNCSs). These sweeteners are commonly used as sugar substitutes, especially in the context of weight control and reducing the negative health effects associated with excessive sugar consumption. Their presence in food and beverages has increased significantly over the last three decades [18]. This study is the first of its kind in Poland to analyze the content of LNCSs in both products with and without added sugar. It was found that 33.9% of the products without added sugar and 20% of the products with added sugar contained LNCSs, which indicates significant differences between these categories. These results are partially consistent with data obtained in other countries. An analysis of 4539 products available in a large supermarket in Brazil showed the presence of LNCSs in 13.3% of products [19], while in Chile, their share was as high as 55.5% [20]. These differences may be due to different food markets and analytical methods, in particular the omission of some sweeteners in the studies from Brazil and Chile. An additional analysis of trends in the use of LNCSs in soft drinks showed that intense sweeteners were present in 25.7% of beverages without added sugar and 50.7% of beverages with added sugar. Comparison of our own Polish results with published data indicates that the share of LNCSs in soft drinks on the Polish market is relatively high compared to that in Slovenia (15.5%) [12], New Zealand (8.7%) and Australia (2.3%) [5] but similar to the results obtained for South America (>40%) [20], the USA (23.9%) [5] and Spain (39.2%) [18]. On the Slovenian market, most beverages contained intense sweeteners, while the presence of polyols was negligible [12]. In the case of our own research, intensive sweeteners also dominated in the group of beverages, while taking into account all of the product groups analyzed, it was shown that in the case of products without added sugar, the most commonly used group of sweeteners was polyols, and in the case of products with added sugar, intensive artificial sweeteners were most common. The analysis of the content of sweeteners showed that 74.6% of products without added sugar did not contain any sweeteners, while in the case of products with added sugar, 40.3% contained only one sweetener. In contrast, research on the Slovenian market showed that most of the analyzed beverages contained more than one sweetener [12]. These differences may suggest differences in consumer preferences and production strategies in individual countries, which emphasizes the need for further research on the sweetener market in a global context.

Studies have shown that the most commonly used sweetener from the LNCS group in the products analyzed on the Polish market is sucralose, which is characterized by high chemical stability, no aftertaste and intense sweetness, approximately 600 times greater than that of sucrose [18]. Sucralose also dominated in studies conducted in Spain and Brazil, where acesulfame K and aspartame were most common [18,19]. Additionally, studies in Ireland and Italy and updated studies from Spain have also confirmed the most common use of sucralose and acesulfame K [18,21,22,23]. In the United States and one study from Hong Kong, it was shown that the most common LNCSs were aspartame and sucralose, which shows their wide use on the global market [15,18,24]. Additionally, another study conducted in Hong Kong showed that the main sweeteners were sorbitols, sucralose and acesulfame K, which indicates similar trends also in Asia [25]. In Slovenia, the analysis of the beverage market in 2017-2019 showed changing preferences in the use of sweeteners, with acesulfame K, aspartame and cyclamine dominating in 2017, while in 2019, the most commonly used LNCSs were acesulfame K, sucralose and aspartame [12]. Sucralose deserves special attention due to its prevalence in food products worldwide. Its chemical stability makes it suitable for a variety of applications, including low-pH and high-temperature conditions [6,12,18]. However, recent studies indicate that during heat treatment of sucralose, degradation products such as chloropropanols may form, which may have potentially toxic effects [26]. Unlike many sweeteners, sucralose does not leave a bitter aftertaste, which further increases its popularity [6,12,18]. In turn, acesulfame K, although widely used, may leave a bitter aftertaste at higher concentrations, which leads to its combination with other sweeteners to obtain a better taste profile [18]. Despite the established acceptable daily intakes (ADIs) for sucralose and acesulfame K [27,28,29], it is recommended to consciously limit their consumption due to the potential health effects associated with their excessive use [27]. In light of these concerns, the World Health Organization (WHO) issued recommendations in 2023 advising against the use of low- and zero-calorie sweeteners as a strategy for weight management or the prevention of noncommunicable diseases. The WHO has emphasized emerging evidence suggesting a potentially increased risk of type 2 diabetes, cardiovascular disease and all-cause mortality associated with their long-term use. Instead, the report suggests a dietary approach involving the selection of unsweetened foods or those containing naturally occurring sugars, such as those found in fruit [30].

In this analysis of the Polish market, it was observed that in products containing added sugar, the most common added sugars were sucrose and glucose syrup. This result is consistent with the research conducted in Australia cited by Samaniego-Vaesken et al., which showed that sucrose, glucose syrup, maple syrup, maltodextrin and glucose/dextrose dominated in a wide group of packaged products, covering 5744 analyzed products [18,31]. Research conducted in Spain, on the other hand, showed that sucrose, glucose-fructose syrup and caramel were the most commonly used forms of added sugar in the analyzed group of food products [18]. Differences in the type of sugars added to products in individual countries may result from both local consumer preferences and the availability of raw materials, which affects the specificity of food products in different markets. However, despite some regional differences, the research results indicate important similarities—in particular, the prevalence of sucrose, which remains the primary sweetening ingredient in different countries. The results obtained in our study are therefore a reflection of broader trends, confirming the central role of sucrose in the composition of food products on the Polish market.

Studies have shown significant differences in the occurrence of polyols between sugar-containing and sugar-free products. Polyols were significantly more often used in products without added sugar. Their presence was detected in the largest percentage of products from the group of sweets and snacks. Their widespread use as sugar substitutes reflects their technological and functional relevance in food production. Although the scientific literature describes both potential benefits and possible adverse effects of polyol consumption, this topic remains a subject of debate [32,33,34,35,36,37,38,39,40,41,42,43]. Results from systematic reviews suggest that the effects of low-calorie sweeteners, including polyols, on metabolism and the gut microbiota may vary depending on the type of substance and the study design [34,35,36,37,38,39,40]. Therefore, it is particularly important to properly inform consumers about the possible effects of their consumption. According to the applicable European Union regulations, the labels of products containing polyols must include a warning: “excessive consumption may induce a laxative effect” [17]. The results of our own analysis confirm the importance of polyols as sugar substitutes on the Polish market and emphasize their growing role in products without added sugar, which is important for shaping dietary and health alternatives. In accordance with Regulation (EC) No. 1333/2008, the use of sweeteners in food is strictly regulated—depending on the product category, their maximum permissible levels are specified, or the “quantum satis” principle is applied [17]. At the national level, the Regulation of the Minister of Health of 22 November 2010 is also in force, which specifies the conditions for the use of additives in Poland [44]. The presence of sweeteners must also be appropriately marked, in accordance with Regulation (EU) No. 1169/2011—e.g., in the case of aspartame, a warning about the source of phenylalanine is required [45]. However, the labels of the products analyzed in Poland do not have to include information on the exact concentrations of sweeteners used, which is a significant limitation in the assessment of the potential level of consumer exposure and highlights the need for further research on the quantitative monitoring of these ingredients.

According to studies conducted by Samaniego-Veasken et al. in Spain, steviol glycosides have limited use in the soft drink industry, constituting only 1% of the analyzed samples [23]. In our own studies, their presence was observed in 1.8% of sugar-free beverages and in 17.4% of sweetened beverages, which indicates significant differences in their use depending on the type of product. In the context of dairy products, Samaniego-Vaesken et al. found steviol glycosides in only 4% of samples [23], and our own studies did not show their presence in this category, which suggests their rare use in milk and dairy products. The analysis of cereal flakes also did not reveal the presence of steviol glycosides in either study, which indicates their limited use in this context, both in Poland and Spain [23]. In addition, studies by Xiao Luo et al. have shown that stevia can successfully replace up to 50% of the sucrose in food products, with minimal effect on their texture and significantly lowering their glycemic index [46]. However, it should be noted that one of the limitations associated with its use is the occurrence of an undesirable aftertaste, which may affect the acceptance of these substances by consumers [46]. Despite these limitations, steviol glycosides have been subjected to extensive studies, which have confirmed their properties as effective sugar substitutes and their safety of use in the food industry [46,47,48,49]. In the context of the growing interest in healthy alternatives to sugar, the results of the analysis indicate the potential of steviol glycosides for creating products with a reduced calorie content, which may be of significant importance for health and nutrition strategies.

The differences identified in the use of sweeteners depending on the labeling of products as “with added sugar” and “without added sugar” indicate the different approaches of manufacturers to the formulation of food products. The more frequent use of polyols and sucralose in products without added sugar reflects their technological role as sugar substitutes, allowing the sweetness and structure of the product to be maintained. In turn, the presence of intense sweeteners in some products with added sugar may indicate attempts to partially reduce their sugar content or improve their taste. Despite the applicable limits on the use of sweeteners in individual products, the practice of combining several different LNCSs into one recipe may result in difficulties in assessing the total exposure to the consumer, especially in cases of repeated consumption of different products containing the same ingredients. The lack of an obligation to quantitatively declare the content of individual LNCSs on labels significantly limits the possibilities to assess their consumption in the population and poses a challenge in the context of monitoring and potential risk assessments. In the context of the increasing incidence of obesity and type 2 diabetes, products without added sugar—containing LNCSs—are increasingly considered as an alternative with reduced energy value in public health activities [50,51,52,53]. For these reasons, further action is needed to improve the clarity of labeling, as is research combining product data with consumption information to allow for more precise estimates of actual exposure.

## 5. Conclusions

This analysis of the composition of food products available on the Polish market showed that most of them did not contain low-calorie or non-calorie sweeteners (LNCSs). However, their presence was noticeable in certain product categories, especially those labeled as “without added sugar”. The most commonly used sugar substitutes in such products were polyols and sucralose. In contrast, products containing sugar were characterized by the rare use of LNCSs, and the main sweetening ingredients were sucrose and glucose syrup.

Due to the lack of an obligation to quantitatively mark the contents of LNCSs on labels, it is not possible to estimate the actual levels of their consumption by consumers based only on data from manufacturers’ declarations. This information gap limits the possibility to assess exposure and makes it difficult to monitor the use of sweeteners in the diet. Potential overlaps in these ingredients between different products may lead to cumulative levels of consumption that are difficult to estimate, even when complying with the applicable regulations.

As this study was based solely on the label analysis, the motivations of the producers or the strategies behind the selection of specific sweeteners were not taken into account. Additionally, although labels are regulated, differences in the way ingredients are declared may affect the unambiguous interpretation of the data. Therefore, such analyses could be complemented by qualitative studies, including industry interviews or reviews of marketing practices. Due to the differences in the LNCS use practices between countries, resulting from, among other things, different legal regulations, consumer preferences and producer strategies, further comparative international studies are also necessary. This will allow for a fuller understanding of the scale and nature of LNCS use in food and may also provide a basis for the development of more coherent and effective regulatory strategies at a European and global scale. It is worth noting that the results presented in this article are preliminary data on the use of low-calorie and non-calorie sweeteners, but their analysis should be expanded to include longitudinal and experimental studies. Such studies would allow for more precise determination of how the use of sweeteners affects long-term dietary behavior and consumer health.

## Figures and Tables

**Table 1:** The amount of sweeteners in the products.

		Score	
No.	Criterion	0	1
1.	Presence of added sucrose	NO	YES
2.	Presence of added fructose	NO	YES
3.	Presence of added glucose	NO	YES
4.	Presence of added lactose	NO	YES
5.	Presence of added lactase	NO	YES
6.	Presence of added maltodextrin	NO	YES
7.	Presence of added isomaltulose	NO	YES
8.	Presence of added sugar	NO	YES
9.	Presence of added glucose syrup	NO	YES
10.	Presence of added sucrose syrup	NO	YES
11.	Presence of added maltose syrup	NO	YES
12.	Presence of added glucose–fructose syrup	NO	YES
13.	Presence of added rice syrup	NO	YES
14.	Presence of added date syrup	NO	YES
15.	Presence of added caramel syrup	NO	YES
16.	Presence of added malt extract	NO	YES
17.	Presence of added honey	NO	YES
18.	Presence of added maple syrup	NO	YES
19.	Presence of added agave syrup	NO	YES
20.	Presence of added molasses	NO	YES
21.	Presence of added acesulfame K	NO	YES
22.	Presence of added aspartame	NO	YES
23.	Presence of added cyclamic acid and/or its salt: sodium and calcium	NO	YES
24.	Presence of added saccharin and its salts: sodium, potassium and calcium	NO	YES
25.	Presence of added sucralose	NO	YES
26.	Presence of added thaumatin	NO	YES
27.	Presence of added neohesperidin DC	NO	YES
28.	Presence of added steviol glycosides	NO	YES
29.	Presence of added neotame	NO	YES
30.	Presence of added aspartame and/or acesulfame salt	NO	YES
31.	Presence of added sorbitol and/or sorbitol syrup	NO	YES
32.	Presence of added mannitol	NO	YES
33.	Presence of added xylitol	NO	YES
34.	Presence of added erythritol	NO	YES
35.	Presence of added maltitol and/or maltitol syrup	NO	YES
36.	Presence of added lactitol	NO	YES
37.	Presence of added isomalt	NO	YES

**Table 2:** A percentage comparison of the products in terms of the presence of selected groups of sweeteners.

	Presence of Low- and No-Calorie Sweeteners	*p*	Presence of Intense Artificial Sweeteners	*p*	Presence of Steviol Glycosides	*p*	Presence of Polyols	*p*
Products without added sugar (N = 744)	33.9%	**<0.0001**	16.8%	**0.005**	4.2%	**0.082**	22.9%	**<0.0001**
Products with added sugar (N = 534)	20.0%		11.2%		2.4%		6.7%	

Note: Statistically significant differences (*p* < 0.05) are shown in bold.

**Table 3:** A comparison of the products in terms of the presence of intense artificial sweeteners, steviol glycosides and polyols, divided into established categories.

Product Group		Presence of Intensive Artificial Sweeteners	*p*	Presence of Steviol Glycosides	*p*	Presence of Polyols	*p*
Desserts	No added sugar (N = 81)	53.1% (N = 43)	**<0.0001**	6.2% (N = 5)	0.0849	13.6% (N = 11)	**0.035**
	With added sugar (N = 46)	0% (N = 0)		0% (N = 0)		2.2% (N = 1)	
Milk and dairy products	No added sugar (N = 10)	10% (N = 1)	**0.01**	0% (N = 0)	-	0% (N = 0)	-
	With added sugar (N = 64)	0% (N = 0)		0% (N = 0)		0% (N = 0)	
Drinks	No added sugar (N = 109)	25.7% (N = 28)	**0.0007**	1.8% (N = 2)	**0.0002**	1.8% (N = 2)	0.6279
	With added sugar (N = 69)	50.7% (N = 35)		17.4% (N = 12)		2.9% (N = 2)	
Cereal flakes	No added sugar (N = 35)	5.7% (N = 2)	0.0661	0% (N = 0)	-	28.6% (N = 10)	**<0.0001**
	With added sugar (N = 58)	0% (N = 0)		0% (N = 0)		0% (N = 0)	
Breath-fresheners	No added sugar (N = 8)	100% (N = 8)	**<0.0001**	0% (N = 0)	-	100% (N = 8)	**<0.0001**
	With added sugar (N = 9)	0% (N = 0)		0% (N = 0)		0% (N = 0)	
Sweets and snacks	No added sugar (N = 255)	4.7% (N = 12)	**0.0016**	7.8% (N = 20)	**<0.0001**	42.8% (N = 109)	**<0.0001**
	With added sugar (N = 207)	0% (N = 0)		0% (N = 0)		15.9% (N = 33)	
Vegetables, fruits and their products	No added sugar (N = 246)	13% (N = 32)	**0.0002**	1.6% (N = 4)	0.7973	12.2% (N = 30)	**0.0034**
	With added sugar (N = 81)	30.9% (N = 25)		1.2% (N = 1)		1.2% (N = 1)	

Note: Statistically significant differences (*p* < 0.05) are shown in bold.

## Data Availability

The original contributions presented in this study are included in the article. Further inquiries can be directed to the corresponding author.

## Abbreviations

The following abbreviations are used in this manuscript:

LNCSs	Low- and No-Calorie Sweeteners
ADI	Acceptable Daily Intake

## References

[1] Gauthier E., Milagro F.I., Navas-Carretero S. (2024). Effect of low-and non-calorie sweeteners on the gut microbiota: A review of clinical trials and cross-sectional studies. Nutrition.

[2] Andersen S.S.H., Zhu R., Kjølbæk L., Raben A. (2023). Effect of Non- and Low-Caloric Sweeteners on Substrate Oxidation, Energy Expenditure, and Catecholamines in Humans—A Systematic Review. Nutrients.

[3] Maluly H.D.B., Johnston C., Giglio N.D., Schreiner L.L., Roberts A., Abegaz E.G. (2020). Low- and No- Calorie Sweeteners (LNCS): Critical evaluation of their safety and health risks. Food Sci. Technol..

[4] Abbeele P.V., Poppe J., Deyaert S., Laurie I., Gravert T.K.O., Abrahamsson A., Baudot A., Karnik K., Risso D. (2023). Low-no-calorie sweeteners exert marked compound-specific impact on the human gut microbiota ex vivo. Int. J. Food Sci. Nutr..

[5] Dunford E.K., Taillie L.S., Miles D.R., Eyles H., Tolentino-Mayo L., Ng S.W. (2018). Non-Nutritive Sweeteners in the Packaged Food Supply—An Assessment across 4 Countries. Nutrients.

[6] Mora M.R., Dando R. (2021). The sensory properties and metabolic impact of natural and synthetic sweeteners. Compr. Rev. Food Saf..

[7] Te Morenga L., Mallard S., Mann J. (2012). Dietary sugars and body weight: Systematic review and meta-analyses of randomised controlled trials and cohort studies. BMJ.

[8] González-Rodríguez M., Redruello-Requejo M., Samaniego-Vaesken M.L., Montero-Bravo A., Puga A.M., Partearroyo T., Varela-Moreiras G. (2021). Low- and No-Calorie Sweetener (LNCS) Presence and Consumption among the Portuguese Adult Population. Nutrients.

[9] Hafner E., Pravst I. (2021). The Sharp Rise in the Use of Low- and No-Calorie Sweeteners in Non-Alcoholic Beverages in Slovenia: An Update Based on 2020 Data. Front. Nutr..

[10] Barraj L., Bi X., Tran N. (2021). Screening level intake estimates of low and no-calorie sweeteners in Argentina, Chile, and Peru. Food Addit. Contam. Part A.

[11] Orku S.E., Suyen G., Bas M. (2022). The effect of regular consumption of four low- or no-calorie sweeteners on glycemic response in healthy women: A randomized controlled trial. Nutrition.

[12] Hafner E., Hribar M., Hristov H., Kušar A., Žmitek K., Roe M., Pravst I. (2021). Trends in the Use of Low and No-Calorie Sweeteners in Non-Alcoholic Beverages in Slovenia. Foods.

[13] Redruello-Requejo M., González-Rodríguez M., Samaniego-Vaesken M.D.L., Montero-Bravo A., Partearroyo T., Varela-Moreiras G. (2021). Low-and no-calorie sweetener (LNCS) consumption patterns amongst the Spanish adult population. Nutrients.

[14] Bielaszka A., Kardas M., Kiciak A., Szczepanska E., Grajek M., Jastrzebska A., Kardas J., Grochowska-Niedworok E. (2016). Wykorzystanie stewii jako zamiennika cukru przez osoby dorosłe. Bromat Chem. Toksykol..

[15] O B.Y.S., Coyle D.H., Dunford E.K., Wu J.H.Y., Louie J.C.Y. (2021). The Use of Non-Nutritive and Low-Calorie Sweeteners in 19,915 Local and Imported Pre-Packaged Foods in Hong Kong. Nutrients.

[16] Barraj L., Scrafford C., Bi X., Tran N. (2021). Intake of low and no-calorie sweeteners (LNCS) by the Brazilian population. Food Addit. Contam. Part A.

[17] European Parliament and Council (2008). Regulation (EC) No 1333/2008 of the European Parliament and of the Council of 16 December 2008 on food additives. Off. J. Eur. Union.

[18] Samaniego-Vaesken M.L., Ruiz E., Partearroyo T., Aranceta-Bartrina J., Gil A., Gonzalez-Gross M., Ortega R.M., Serra-Majem L., Varela-Moreiras G. (2018). Added Sugars and Low- and No-Calorie Sweeteners in a Representative Sample of Food Products Consumed by the Spanish ANIBES Study Population. Nutrients.

[19] Figueiredo L.S., Scapin T., Fernandes A.C., Proença R.P.C. (2017). Where are the low-calorie sweeteners? An analysis of the presence and types of low-calorie sweeteners in packaged foods sold in Brazil from food labelling. Public Health Nutr..

[20] Sambra V., López-Arana S., Cáceres P., Abrigo K., Collinao J., Espinoza A., Valenzuela S., Carvajal B., Prado G., Peralta R. (2020). Overuse of Non-caloric Sweeteners in Foods and Beverages in Chile: A Threat to Consumers’ Free Choice?. Front. Nutr..

[21] Buffini M., Goscinny S., Van Loco J., Nugent A.P., Walton J., Flynn A., Gibney M.J., McNulty B.A. (2018). Dietary intakes of six intense sweeteners by Irish adults. Food Addit. Contam. Part A.

[22] Le Donne C., Mistura L., Goscinny S., Janvier S., Cuypers K., D’Addezio L., Sette S., Catasta G., Ferrari M., Piccinelli R. (2017). Assessment of dietary intake of 10 intense sweeteners by the Italian population. Food Chem. Toxicol..

[23] Samaniego-Vaesken M.L., Partearroyo T., Cano A., Urrialde R., Varela-Moreiras G. (2019). Novel database of declared low- and no-calorie sweeteners from foods and beverages available in Spain. J. Food Compos. Anal..

[24] Sylvetsky A.C., Rother K.I. (2016). Trends in the consumption of low-calorie sweeteners. Physiol. Behav..

[25] Yen S.H.Y., Barrett E., Coyle D.H., Wu J.H.Y., Louie J.C.Y. (2024). The distribution and co-occurrence of food additives in pre-packaged foods in Hong Kong. Food Control.

[26] Eisenreich A., Gurtler R., Schafer B. (2020). Heating of food containing sucralose might result in the generation of potentially toxic chlorinated compounds. Food Chem..

[27] Kujałowicz A. (2022). Charakterystyka wybranych cukrów i słodzików. Tutoring Gedanensis.

[28] Shaher S.A.A., Mihailescu D.F., Amuzescu B. (2023). Aspartame Safety as a Food Sweetener and Related Health Hazards. Nutrients.

[29] EFSA Panel on Food Additives and Nutrient Sources added to Food (ANS) (2011). Scientific Opinion on the re-evaluation of butylated hydroxyanisole–BHA (E 320) as a food additive. EFSA J..

[30] World Health Organization (2023). Use of Non-Sugar Sweeteners: WHO Guideline Summary.

[31] Probst Y.C., Dengate A., Jacobs J., Louie J.C., Dunford E.K. (2017). The major types of added sugars and non-nutritive sweeteners in a sample of Australian packaged foods. Public Health Nutr..

[32] Hu X., Song L., Yang Y., Wang L., Li Y., Miao M. (2021). Biosynthesis, structural characteristics and prebiotic properties of maltitol-based acceptor products. J. Funct. Foods.

[33] Shen P., Walker G.D., Yuan Y., Reynolds C., Reynolds E.C. (2017). Polyols and remineralisation of enamel subsurface lesions. J. Dent..

[34] Godswill A.C., Kate E.C. (2019). Current developments in sugar alcohols: Chemistry, nutrition, and health concerns of sorbitol, xylitol, glycerol, arabitol, inositol, maltitol, and lactitol. Int. J. Adv. Acad. Research. Sci. Technol. Eng..

[35] Plaza-Diaz J., Pastor-Villaescusa B., Rueda-Robles A. (2020). Plausible Biological Interactions of Low- and Non-Calorie Sweeteners with the Intestinal Microbiota: An Update of Recent Studies. Nutrients.

[36] Ruiz-Ojeda F.J., Plaza-Diaz J., Sáez-Lara M.J., Gil A. (2019). Effects of Sweeteners on the Gut Microbiota: A Review of Experimental Studies and Clinical Trials. Adv. Nutr..

[37] Saraiva A., Carrascosa C., Raheem D., Ramos F., Raposo A. (2020). Maltitol: Analytical Determination Methods, Applications in the Food Industry, Metabolism and Health Impacts. Int. J. Env. Res. Public Health.

[38] Lenhart A., Chey W.D. (2017). A Systematic Review of the Effects of Polyols on Gastrointestinal Health and Irritable Bowel Syndrome. Adv. Nutr..

[39] Wan Z., Khubber S., Dwivedi M., Misra N.N. (2021). Strategies for lowering the added sugar in yogurts. Food Chem..

[40] Janakiram C., Kumar C.V.D., Joseph J. (2017). Xylitol in preventing dental caries: A systematic review and meta analyses. J. Nat. Sci. Biol. Med..

[41] Szynal K., Polaniak R., Górski M., Grajek M., Ciechowska K., Grochowska-Niedworok E. (2021). Processed Food and Food Additives in the Context of Dysbiosis and Its Health Consequences. Postępy Mikrobiol..

[42] Nettleton J.E., Reimer R.A., Shearer J. (2016). Reshaping the gut microbiota: Impact of low calorie sweeteners and the link to insulin resistance?. Physiol. Behav..

[43] Lobach A.R., Roberts A., Rowland I.A. (2019). Assessing the in vivo data on low/no-calorie sweeteners and the gut microbiota. Food Chem. Toxicol..

[44] (2010). Rozporządzenie Ministra Zdrowia z Dnia 22 Listopada 2010 r. w Sprawie Dozwolonych Substancji Dodatkowych. https://isap.sejm.gov.pl/isap.nsf/DocDetails.xsp?id=WDU20102321525.

[45] Regulation (EU) No 1169/2011 of the European Parliament and of the Council of 25 October 2011 on the Provision of Food Information to Consumers, Amending Regulations (EC) No 1924/2006 and (EC) No 1925/2006 of the European Parliament and of the Council, and Repealing Commission Directive 87/250/EEC, Council Directive 90/496/EEC, Commission Directive 1999/10/EC, Directive 2000/13/EC of the European Parliament and of the Council, Commission Directives 2002/67/EC and 2008/5/EC and Commission Regulation (EC) No 608/2004 Text with EEA Relevance. https://eur-lex.europa.eu/eli/reg/2011/1169/oj/eng.

[46] Luo X., Arcot J., Gill T., Louie J.C.Y., Rangan A. (2019). A review of food reformulation of baked products to reduce added sugar intake. Trends Food Sci. Technol..

[47] Talevi A. (2022). Potential medicinal effects and applications of stevia constituents. Phytochem. Rev..

[48] Samuel P., Ayoob K.T., Magnuson B.A., Wölwer-Rieck U., Jeppesen P.B., Rogers P.J., Rowland I., Mathews R. (2018). Stevia Leaf to Stevia Sweetener: Exploring Ist Science, Benefits, and Future Potential. J. Nutr..

[49] Peteliuk V., Rybchuk L., Bayliak M., Storey K.B., Lushchak O. (2021). Natural sweetener stevia rebaudiana: Functionalities, health benefits and potential risks. EXCLI J..

[50] Fattore E., Botta F., Agostoni C., Bosetti C. (2017). Effects of free sugars on blood pressure and lipids: A systematic review and meta-analysis of nutritional isoenergetic intervention trials. Am. J. Clin. Nutr..

[51] Prinz P. (2019). The role of dietary sugars in health: Molecular composition or just calories?. Eur. J. Clin. Nutr..

[52] Grembecka M. (2015). Sugar alcohols—Their role in the modern world of sweeteners: A review. Eur. Food Res. Technol..

[53] Miller P.E., Perez V. (2014). Low-calorie sweeteners and body weight and composition: A meta-analysis of randomized controlled trials and prospective cohort studies. Am. J. Clin. Nutr..

